# Refractory Ventricular Arrhythmias in Thyrotoxic Periodic Paralysis: An Uncommon Presentation of Cardiogenic Shock

**DOI:** 10.7759/cureus.87801

**Published:** 2025-07-12

**Authors:** Faizan Ahmed, Phillip Lindsey, Aneeta Bhatia

**Affiliations:** 1 Anesthesiology, University of Louisville School of Medicine, Louisiville, USA; 2 Anesthesiology, University of Louisville Hospital, Louisville, USA

**Keywords:** cardiac arrythmia, cardiothoracic anesthesia, endocrine abnormality, pulmonary critical care, va-ecmo

## Abstract

Thyrotoxic periodic paralysis (TPP) is a rare complication of hyperthyroidism, often seen in young males, characterized by acute flaccid paralysis due to intracellular potassium shifts. While most cases present with isolated muscle weakness, progression to thyroid storm with life-threatening arrhythmias and multiorgan failure is exceedingly rare. We present the case of a previously healthy 23-year-old male who arrived with profound weakness and was found to have severe hypokalemia and suppressed thyroid-stimulating hormone (TSH). Shortly after arrival, he developed ventricular fibrillation and suffered prolonged cardiac arrest, requiring nearly 90 minutes of cardiopulmonary resuscitation. He was placed on extracorporeal membrane oxygenation (ECMO) for refractory cardiogenic shock. His hospital course was further complicated by rhabdomyolysis, acute kidney injury requiring renal replacement therapy, and distal limb ischemia due to femoral cannulation, ultimately necessitating left above-knee amputation and right upper extremity fasciotomy. This case highlights the catastrophic potential of TPP and thyroid storm, the role of early ECMO support in refractory thyrotoxic arrhythmias, and the vascular risks associated with peripheral ECMO cannulation in the critically ill. Prompt recognition and aggressive support remain the cornerstone of survival in endocrine-driven cardiopulmonary collapse.

## Introduction

Thyrotoxic periodic paralysis (TPP) is a rare but potentially devastating manifestation of thyrotoxicosis, characterized by sudden episodes of muscle weakness and severe hypokalemia due to intracellular potassium shifts. While TPP is well-documented in East Asian populations, its incidence in non-Asian patients is often underrecognized, contributing to diagnostic delays and poor outcomes [1]. The condition is typically reversible with appropriate correction of the underlying thyroid dysfunction and electrolyte derangements. However, in extreme cases, TPP may progress to thyroid storm, a life-threatening hypermetabolic crisis with cardiovascular collapse, malignant arrhythmias, and multiorgan failure.

Cardiac involvement in TPP, though uncommon, presents a significant risk. The profound hypokalemia seen in TPP alters myocardial excitability, predisposing patients to ventricular tachyarrhythmias and sudden cardiac arrest. The combination of hyperthyroidism, adrenergic surge, and electrolyte disturbance forms a perfect substrate for electrical instability [2]. Management in such cases is further complicated by the hemodynamic instability and need for advanced circulatory support, such as extracorporeal membrane oxygenation (ECMO).

This report details the case of a previously healthy young male who presented with classic signs of TPP but rapidly deteriorated into cardiac arrest due to refractory ventricular arrhythmias, ultimately requiring ECMO support. The case is further complicated by vascular compromise from femoral cannulation, leading to limb loss. Through this case, we aim to underscore the importance of early recognition of TPP, explore the pathophysiologic mechanisms underlying its cardiac manifestations, and discuss the role and risks of ECMO in endocrine-driven cardiogenic shock.

## Case presentation

A previously healthy 23-year-old male presented to the emergency department with complaints of intermittent weakness, muscle cramps, and transient numbness in all four extremities. He had reported unintentional weight loss over several weeks. According to the family, he had also experienced tremors, diarrhea, anxiety, and insomnia, concerning for underlying thyrotoxicosis. Notably, he had sustained a minor back injury 1.5 weeks prior, followed by worsening cramping and episodic leg numbness.

**Figure 1:**
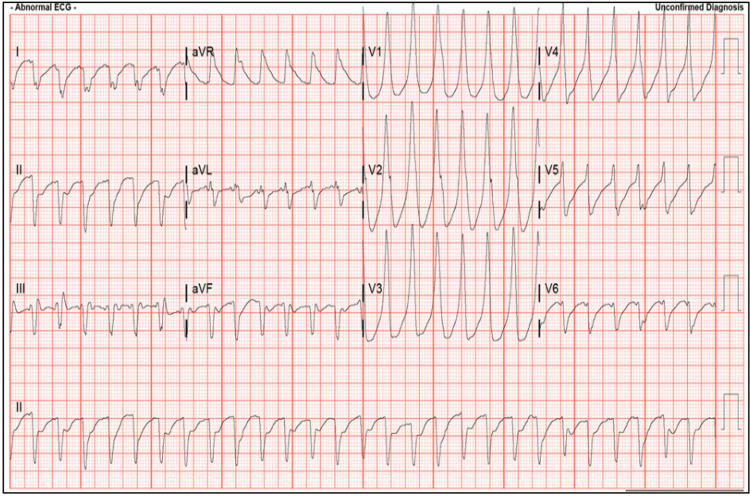
Polymorphic refractory ventricular tachycardia secondary to thyrotoxic periodic paralysis in the emergency department.

While in the emergency department, the patient developed polymorphic ventricular tachycardia that degenerated into ventricular fibrillation, as seen in Figure 1. He suffered a prolonged cardiac arrest requiring approximately 90 minutes of advanced cardiac life support (ACLS), including multiple defibrillations, amiodarone, esmolol, and vasopressor support with high-dose epinephrine, norepinephrine, and vasopressin. Return of spontaneous circulation (ROSC) was achieved, and the patient was intubated. Imaging was concerning for ARDS and pulmonary contusions, likely secondary to continuous compressions. He was then subsequently transferred to a regional cardiothoracic intensive care unit for veno-arterial-venous (VAV) extracorporeal membrane oxygenation (ECMO) due to refractory cardiogenic and distributive shock as well as for pulmonary support.

Initial laboratory studies revealed a potassium level of 1.0 mEq/L, pH < 6.7, and thyroid-stimulating hormone (TSH) < 0.01 mIU/L, with elevated free T4 (2.93 ng/dL), confirming the diagnosis of TPP in the setting of thyroid storm. Thyroid peroxidase antibodies were positive, consistent with Graves’ disease. A transesophageal echocardiogram (TEE) showed preserved biventricular function.

VAV ECMO was initiated via left femoral vein drainage, left femoral artery arterial return, and right internal jugular venous return. Continuous renal replacement therapy (CRRT) was started for acute oliguric renal failure and severe lactic acidosis. Endocrinology initiated antithyroid therapy with propylthiouracil, cholestyramine, and stress-dose hydrocortisone; potassium iodide was withheld due to recent iodinated contrast and amiodarone exposure. Beta-blockers were initially deferred due to hemodynamic instability and later introduced using propranolol.

Furthermore, the patient developed acute left lower extremity ischemia secondary to arterial cannulation, resulting in compartment syndrome requiring emergent fasciotomy. Due to irreversible ischemic injury, the patient underwent left above-knee amputation (AKA). A rising CK and mottled skin appearance prompted circuit reconfiguration to veno-arterial (VA) configuration with right axillary arterial return. The following day, he developed absent pulses in the right upper extremity. Right axillary exploration and stent placement were performed, restoring perfusion. Right upper extremity fasciotomy was performed the following day for compartment syndrome.

The patient was initially extubated but required re-intubation due to respiratory fatigue. He was successfully extubated again after demonstrating improved respiratory mechanics and gas exchange. During this period, he underwent debridement of the left lower extremity stump, partial closure of fasciotomy sites, and placement of a wound vacuum-assisted closure (VAC) device. Pulmonary hygiene was maintained with high-flow oxygen support, mucolytics, and incentive spirometry to address bilateral lower lobe atelectasis and evolving ARDS. He subsequently underwent definitive surgical treatment with total thyroidectomy. Postoperative management included levothyroxine replacement and calcium plus vitamin D supplementation for transient hypoparathyroidism.

The patient underwent intensive inpatient rehabilitation focused on mobility following a left AKA and persistent weakness of the right upper extremity. He demonstrated gradual functional improvement and was ultimately discharged to a skilled nursing facility. At the time of discharge, he was neurologically intact, breathing comfortably on room air, with stable hemodynamics and endocrine function. He maintained independent bowel and bladder function and was planned for outpatient follow-up with endocrinology, physical rehabilitation, and prosthetic fitting services.

## Discussion

TPP is a rare but life-threatening neuromuscular complication of hyperthyroidism characterized by episodes of flaccid muscle paralysis and profound hypokalemia. Despite its prevalence of approximately 0.5% among patients with hyperthyroidism in the United States, TPP remains underrecognized, particularly in non-Asian populations [3]. In its most severe form, TPP may precipitate life-threatening cardiac arrhythmias and respiratory failure, as observed in our patient, who suffered a prolonged cardiac arrest due to refractory ventricular fibrillation in the setting of a potassium level of 1.0 mEq/L.

**Figure 2:**
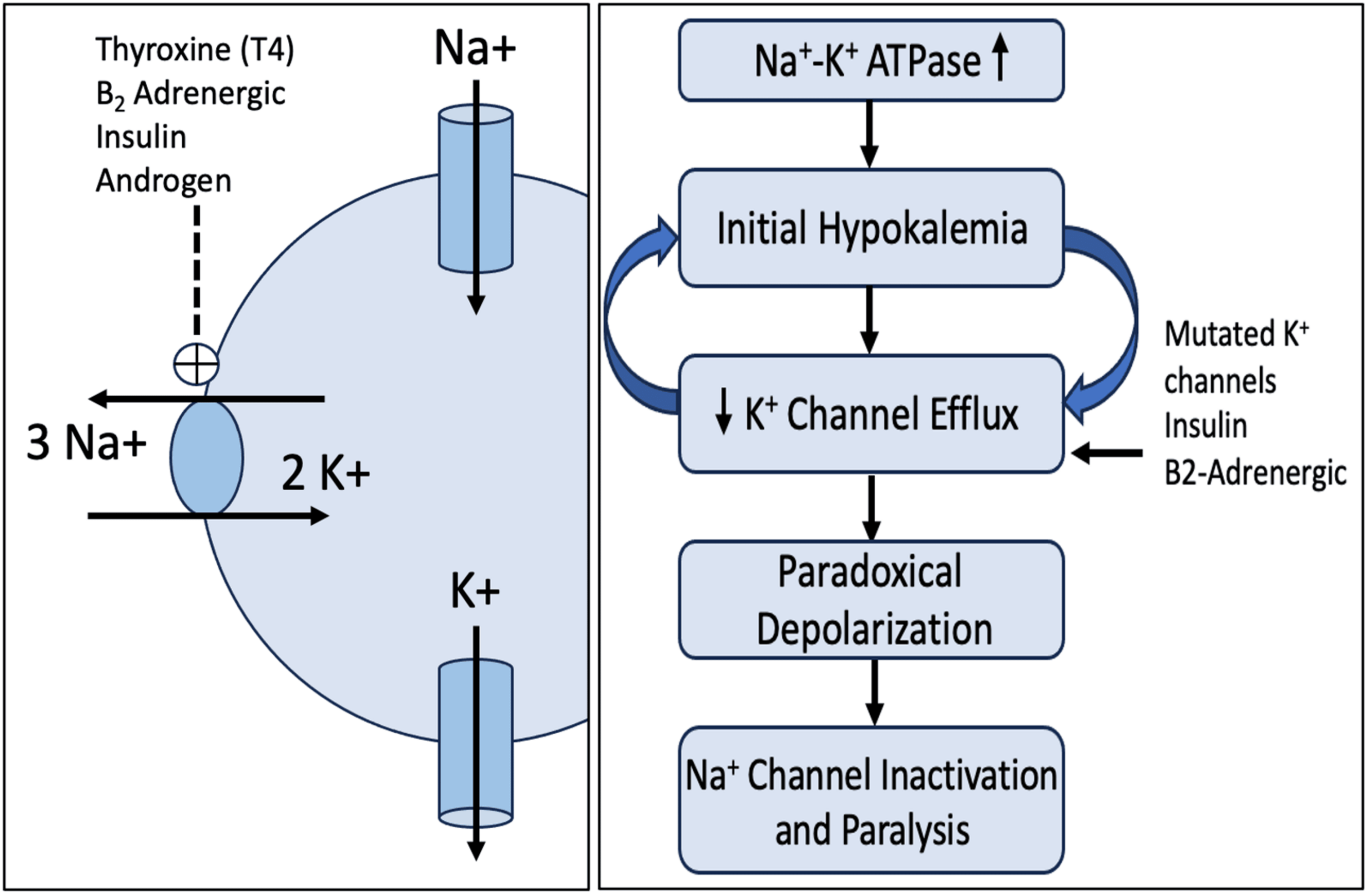
Pathophysiology of thyrotoxic periodic paralysis. Image credit:  Phillip Lindsey.

The pathophysiology of TPP involves a complex interplay between thyroid hormone excess, genetic predisposition, and increased β-adrenergic stimulation. Thyroid hormones upregulate the expression and activity of the Na⁺/K⁺-ATPase pump, particularly in skeletal muscle, leading to an intracellular shift of potassium. This process is potentiated by insulin, catecholamines, and androgens, which further stimulate Na⁺/K⁺-ATPase activity. The result is a precipitous drop in serum potassium despite total body stores being normal or elevated. Compounding this is a suspected channelopathy in susceptible individuals, wherein potassium efflux through skeletal muscle channels is impaired, as seen in Figure 2. The imbalance between inward potassium shift and restricted efflux creates a paradoxical depolarization of muscle membrane potential, rendering Na⁺ channels inactive and ultimately leading to flaccid paralysis [4]. 

In addition to muscle paralysis, this derangement in cellular electrophysiology predisposes to arrhythmogenesis. Hypokalemia reduces repolarization reserve and prolongs the QT interval, increasing the risk of reentrant ventricular arrhythmias. The synergistic effects of thyroid hormone excess, β-adrenergic stimulation, and critical hypokalemia formed the perfect storm in our patient, culminating in cardiac arrest refractory to standard ACLS interventions.

Our patient developed cardiogenic shock requiring initiation of VA-ECMO as a bridge to recovery. While ECMO provides circulatory support during reversible cardiopulmonary failure, it carries significant risks. Femoral artery cannulation is associated with lower extremity ischemia, and our patient required left above-knee amputation and right upper extremity fasciotomy following cannulation-related complications. The 2020 consensus statement by the European Association for Cardio-Thoracic Surgery (EACTS), Extracorporeal Life Support Organization (ELSO), Society of Thoracic Surgeons (STS), and American Association for Thoracic Surgery (AATS) reports that limb ischemia requiring surgical intervention may occur in up to 17% of ECMO cases [5]. A 2014 meta-analysis of 1,866 adult patients showed pooled complication rates of 16.9% for lower extremity ischemia, 10.3% for fasciotomy or compartment syndrome, and 4.7% for amputation [6].

Successful recovery in this case required a multidisciplinary approach. Endocrinology played a critical role in guiding anti-thyroid therapy and postoperative hormone replacement following total thyroidectomy. Surgical teams managed vascular complications, and the critical care team coordinated renal support, ventilator management, and ECMO weaning. Despite significant morbidity, including limb loss, acute kidney injury, and prolonged mechanical ventilation, the patient achieved neurologic recovery and was discharged to rehabilitation with intact cognition and preserved organ function.

## Conclusions

TPP remains a rare but potentially fatal endocrine emergency that can rapidly escalate into cardiopulmonary collapse. In cases of life-threatening arrhythmias and multiorgan failure, early initiation of ECMO can serve as a vital bridge to recovery. However, ECMO itself carries substantial risks, including limb-threatening ischemia. This case highlights the importance of early recognition, aggressive endocrine and critical care management, and a multidisciplinary approach to optimize outcomes in patients with thyroid storm-associated complications.

## References

[1] Verma V, Kumar Y, Kotwal N, Upreti V, Hari Kumar KV, Singh Y, Menon AS (2020). Thyrotoxic periodic paralysis: a retrospective, observational study from India. Indian J Med Res.

[2] Noso S, Babaya N, Hiromine Y (2019). Contribution of Asian haplotype of KCNJ18 to susceptibility to and ethnic differences in thyrotoxic periodic paralysis. J Clin Endocrinol Metab.

[3] Rivas AM, Thavaraputta S, Orellana-Barrios MA, Payne JD, Sotello D, Vinan-Vega M, Lado-Abeal J (2020). Thyrotoxic periodic paralysis and complicated thyrotoxicosis, two presentations of hyperthyroidism with notable differences in their clinical manifestations: an experience from a tertiary care hospital in the United States. Endocr Pract.

[4] Lin SH, Huang CL (2012). Mechanism of thyrotoxic periodic paralysis. J Am Soc Nephrol.

[5] Lorusso R, Whitman G, Milojevic M (2021). 2020 EACTS/ELSO/STS/AATS expert consensus on post-cardiotomy extracorporeal life support in adult patients. Eur J Cardiothorac Surg.

[6] Cheng R, Hachamovitch R, Kittleson M (2014). Complications of extracorporeal membrane oxygenation for treatment of cardiogenic shock and cardiac arrest: a meta-analysis of 1,866 adult patients. Ann Thorac Surg.

